# Factors affecting mode of delivery in a nullipara at term with singleton pregnancy and vertex presentation (NTSV)

**DOI:** 10.12669/pjms.322.9138

**Published:** 2016

**Authors:** Iffat Ahmed, Uzma Chishti, Munazza Akhtar, Humaira Ismail

**Affiliations:** 1 Dr. Iffat Ahmed, Senior Instructor, Department of Obstetrics and Gynaecology, Aga Khan University Hospital, Karachi, Pakistan; 2 Dr. Uzma Chishti, Assistant Professor, Department of Obstetrics and Gynaecology, Aga Khan University Hospital, Karachi, Pakistan; 3 Dr. Munazza Akhtar, Senior Instructor, Department of Obstetrics and Gynaecology, Aga Khan University Hospital, Karachi, Pakistan; 4 Ms. Humaira Ismail, Research Specialist, Department of Obstetrics and Gynaecology, Aga Khan University Hospital, Karachi, Pakistan

**Keywords:** Caesarean section, Induction of labour, Low risk pregnancy, Nulliparous, Singleton pregnancy, vertex presentation, Term pregnancy

## Abstract

**Objective:**

To analyse the factors associated with Caesarean Section (CS) of Nulliparous, Term and Singleton pregnancies with Vertex presentation (NTSV) at a tertiary care hospital.

**Methods:**

In this unmatched retrospective case-control study, 212 NTSV patients were identified through computerized medical record systems; the data was collected through predesigned Performa by reviewing medical record charts. One hundred six CS and spontaneous vaginal deliveries (SVD) were taken as cases and controls.

**Results:**

The mean maternal age of cases (CS) was 26.64 (SD: 3.9) and of controls (SVD) was 26.7(SD: 3.9) years, whereas mean gestational age was 38.66±1.12 and 38.57±0.9 weeks for cases and controls respectively. Ninety per cent of women in the study group were delivered within 10 hours of active labour. Babies that weighed ≤3kg were 45% and >3kg were 55%. The possibility of being high risk was twice more among those delivered by CS. However, it was not statistically significant (p value 0.077). Labour was induced in 38% patients. The Odds of Induction of Labour (IOL) were two times more and delivering at night was three times more amongst CS. The likelihood of labour exceeding 10 hours was four times (81%) if the patient had a CS. Moreover 48% of the babies weighing >3kg were delivered through CS. Maternal age, high risk pregnancies, gender of baby and epidural analgesia were not statistically significant predictors of mode of delivery (MOD) in this study.

**Conclusion:**

Induction of Labour, night time delivery, prolonged labour and birth weight <3kg were found to be associated with the increased CS rate among NTSV. Therefore further research is required in order to address these factors and to reduce the increasing Caesarean Section.

## INTRODUCTION

The caesarean delivery rate has been on steady rise worldwide. It has elevated to 53% in some states of US and globally the rate of caesarean section (CS) in Nulliparous at term and singleton pregnancy with vertex presentation (NTSV) varies widely, from 10.3% to 34.2%.^1^-^4^ There are many factors associated with this rise; like medico legal reasons, scheduling issues, economic pressures, provider and patient-driven medicalization of birth,^4^,^5^ increased induction rates^6^ and a broader perception of caesarean as a safe method of delivery.

In addition to that NTSV exerts a major impact on overall statistics, affecting the mode of delivery in subsequent pregnancies and these groups of parturient should achieve vaginal delivery. Hence, we can say that the mode of first delivery is of prime importance in determining the future obstetrics course.^7^ Therefore, identification and addressing the factors leading to CS among NTSV should be emphasized upon to reduce the overall CS rate.^8^

Increase in maternal age, increased birth weight, gender of baby and the presence of medical conditions such as diabetes and hypertension are strongly associated with the mode of delivery in different studies. Induction of Labour (IOL), delivering at day/night time and the use of analgesics, all influence this increased risk of CS.^1^

This study aims to analyse the factors associated with increasing rate of CS in NTSV at a tertiary care hospital. This is mainly of concern among patients who ended in emergency Caesarean Section after failed attempt at vaginal delivery, as the rate of elective CS in the study population is static and within the bench-mark. Hence only emergency CS were included in the study group.

## METHODS

This unmatched retrospective case-control study was conducted at a tertiary care hospital in Karachi, Pakistan, after the approval of the institute’s ethical review committee. The hospital has one of the largest perinatal and neonatal tertiary care facility and a referral centre to cater high risk pregnancies.

For the study, Nulliparous—patients who never delivered a baby of >24 weeks or >500gms, Term—pregnancies ≥37 completed weeks, Singleton with Vertex presentation (NTSV) were included. Whereas, patients with indication of elective caesarean section or if admitted after delivering at some other facilities were excluded.

The NTSV patients admitted between March 1^st^, 2014 and June 30^th^ 2014, for labour management and delivery were identified through computer based medical record system. Complete data was collected through a predesigned Performa and by reviewing the medical record charts. Patients delivered by CS were taken as case and those delivered vaginally including both, spontaneous and operative vaginal deliveries were considered as control. The sample size was calculated with the help of WHO sample size calculator. The assumptions were proportion of IOL among vaginal delivery 32%, among CS 52%^1^, the level of significance was 5% and the power was taken at 80%. As per calculations, total of 212 patients were included in the study; 106 in each arm, through purposive sampling.

The patient was considered as a booked patient if she had a minimum of three antenatal visits in the third trimester at the study institute. The risk factors were identified including diabetes/GDM, hypertensive disorders, obstetric cholestasis or other medical conditions like anti-phospholipid syndrome, cardiac valvular disease and hypothyroidism. The obstetric risks included fetal growth disorder (IUGR/SGA), pre labour rupture of membranes, assisted conception (ICSI/IVF) and oligohydromnios.

The duration of active labour was categorized as ≤10 hours and > 10 hours. And timing of delivery was divided into; day time from 0800 hours till 2000 hours and night time from 2000-0800 hours. The day time included mainly the working hours and the decisions were mainly taken by more experienced obstetricians as compared to those during night hours.

The departmental policy for the management of spontaneous labour at term is according to standard protocol which includes early amniotomy in active labour and augmentation if progress is <1cm in an hour. The decision of Emergency Lower Segment Caesarean Section (LSCS) was taken on suspicion of fetal distress on Cardiotocography trace before full dilation of cervical os or if the delivery was not imminent even after 10 to 12 hours of active labour.

Labour was usually induced for post-dates after completion of 41 weeks, or for obstetric reasons such as diabetes/GDM, hypertensive disorders, obstetric cholestasis, fetal growth issues and oligohydromnios. According to the departmental induction of labour (IOL) guideline, the method of induction includes insertion of 24 gauge intra-cervical Foley’s catheter for a maximum time period of 10 to 12 hours followed by dinoprostone passary or amniotomy if favoured by bishops scoring. The management of active labour for the induced was the same as for that of spontaneous labour. Any induction before 41 completed weeks, without any obstetric or medical reason was considered as social induction, but on the basis of patients’ and physicians’ convenience.

The progress of labour and the events during labour ward admission were recorded on a standardized partogram by designated staff nurses. The details were then entered into a computerized data base by the labour room resident attending that patient.

### Statistical Analysis

Data was entered twice, checked, validated and analysed by using SPSS 19. Frequencies and percentages (descriptive statistics) were calculated for each study variable including booking status at study institute, risk category (low and high risk pregnancy), spontaneous or induced labour, epidural use during labour, duration of labour category, timing of delivery, and gender and weight of the baby. Initially the mean ± SD of numeric values, including maternal age, gestational age and birth weight of babies were calculated and then frequencies of all variables were reviewed and categorical variables were compared by chi-square test. In addition to that, Multivariable analysis using multiple logistic regression was used to identify factors associated with MOD.

## RESULTS

Two hundred and twelve patients were enrolled during the study. The MOD was in equal ratio of 1:1 (106:106), patients delivered through CS were taken as cases and those through vaginal deliveries as controls. The mean maternal age of cases (CS) was 26.64 (SD:3.9) and of controls (SVD) was 26.7(SD:3.9) years, whereas mean gestational age was 38.66±1.12 and 38.57±0.9 weeks for cases and controls respectively. Majority of the patients (96%) were booked at study institute for antenatal care. Out of all the patients, 120 (57%) were low risk while 43% had either medical or obstetric issues. In 131(62%) patients labour started spontaneously while in 81(38%) labour was induced. Epidural analgesia was administered to 57 (27%) patients.

Ninety per cent patients were delivered within 10 hours of labour. The time range of delivery was broadly divided into two categories of 12 hours each i.e.; day time from 0800 hours to 2000 hours, which mostly includes the routine working hours, and the night time from 2000 hours to 0800 hours. Eighty per cent patients were delivered during day time (from 0800-2000 hours).

Out of a total of 212 babies, 114 (54%) were male and 98 (46%) were female. Mean birth weight was 3.08(SD: 0.43) for cases and 3.1 (SD: 0.3) kg for control group. The babies with birth weight of ≤ 3 kg were 45% and 55% weighed>3 kg. The birth weight ranges from 2.2kg to 4.8 kg. (Table-I)

**Table-I:** The percentage distribution of variables of NTSV patients for mode of delivery, cases LSCS (N=106) and controls SVDs (N=106).

Characteristic	SVDs n(%) mean±SD	LSCS n(%) mean±SD	P value
Maternal age (year)	26.71±4.37	26.64±3.98	
Gestational Age (weeks)	38.57±0.9	38.66±1.12	
Booking Status				
Booked	102 (50)	102 (50)	0.63
Un-booked	4 (50)	4 (50)	
Risk Factors				
Low risk	69(57.5)	51 (42.5)	0.018
High risk	37(40.2)	55 (59.8)	
Miscarriages				
No miscarriage	88 (51.2)	84 (48.8)	0.59
≥1 miscarriages	18(45)	22(55)	
Induction of Labour				
No	76(58.0)	55(42.0)	0.002
Yes	30(37.0)	51(63.0)	
Epidural Analgesia				
No	81(52.3)	74(47.7)	0.176
Yes	25(43.9)	32(56.1)	
Duration of labour				
≤ 10 hours	102(53.4)	89(46.6)	0.002
>10 hours	4(19.0)	17(81.0)	
Delivery Time				
0800-2000hours	93(55.0)	76 (45.0)	0.003
2000-0800 hours	13(30.2)	30(69.8)	
Gender of Baby				
Male	57(53.7)	60(56.6)	0.5
Female	49(46.2)	46(28.3)	
Baby Weight (Kg)	3.1±0.3	3.08±0.48	
≤ 3 Kg	41(43.2)	54(56.8)	0.049
> 3Kg	65(55.6)	52 (44.4)	

When the crude OR was calculated, medical and obstetrical risks were found to be twice as more common in the patients delivered through CS than in those, delivered vaginally. However, it was not statistically significant when adjusted with other factors in a regression model. (p value 0.077)

Induction of labour was significantly associated with MOD. The patients delivered through CS were twice more likely to have induction of labour as compared to those delivered virginally. [Adjusted Odd Ratio (AOR) 1.87; 95% CI: 0.99, 3.54; P value: 0.053].

Seventy per cent of patients delivering at night (0800-2000 hours) had CS, and this was statistically significant. The Odds of delivering at night is 3 times for CS than for vaginal delivery [Adjusted Odd Ratio (AOR) 2.85; 95% CI: 1.33, 6.10; P value: 0.007]

The likelihood of labour to exceed for more than 10 hours is four times (81%) for abdominal delivery than for an SVD. [Adjusted Odd Ratio (AOR) 4.49; 95% CI: 1.33, 15.06; P value: 0.015]. Moreover the chances of CS is 48% if the birth weight of baby is >3kg [AOR 0.52; 95% CI 0.28, 0.096, P value 0.036] (Table-II). The gender of baby was not found to be statistically significant predictors of MOD in this study.

**Table-II:** Comparison of spontaneous and C- Section in NTSV deliveries by, maternal and delivery characteristics with adjusted OR.

Characteristic	LSCS n (%)	Vaginal Delivery n (%)	Crude OR(95%CI)	Adjusted OR (95%CI)	P value
Risk Factors					
Low Risk	51(42.5)	69(57.5)	1	1	
High Risk	55 (59.8)	37 (40.2)	2.01(1.15,3.49)	1.73(0.94,3.18)	0.077
Induction of Labour					
No	55(42.0)	76(58.0)	1	1	
Yes	51(63.0)	30(37.0)	2.34(1.33,4.15)	1.87(0.99,3.54)	0.053
Working TimeRange					
0800-2000 hours	76(45.0)	93(55.0)	1	1	
2000-0800 hours	30(69.8)	13(30.2)	2.82(1.37,5.78)	2.85(1.33,6.10)	0.007
Duration of labour					
≤ 10 hours	89(46.6)	102(53.4)	1	1	
>10 hours	17(81.0)	4(19.0)	4.87(1.58,15.01)	4.49(1.33,15.06)	0.015
Gender of baby					
Male	60(56.6)	57(50.0)	1	1	
Female	46(28.3)	49(50.0)	1.00(0.58,1.71)	0.84(0.46,1.54)	0.592
Baby Weight (Kg)					
≤ 3	54(56.8)	41(43.2)	1	1	
>3	52(44.4)	65(55.6)	0.60(0.35,1.04)	0.52(0.28,0.96)	0.036

## DISCUSSION

The present study showed that the MOD of nulliparous mothers, at term having singleton pregnancy with vertex presentation is significantly influenced by induction of labour, timing of delivery, duration of labour and the birth weight of baby. While maternal age, antenatal booking, having medical or obstetric issues, use of epidural and gender of baby had no association with the MOD in an NTSV pregnancy.

The likelihood of emergency CS is more in induced as compared to spontaneous labours. This finding is consistent with the results of Sharma et al and others.^1^,^6^,^9^ Therefore the indication for IOL should be robust enough to justify abdominal delivery in a nulliparous. It is also recommended that women should be counselled regarding the risks of CS prior to consenting for IOL.

When the crude OR was calculated, medical and obstetrical risks were found to be twice more common in patients delivered through CS than those delivered vaginally. In the present study, the high risk pregnancies were found to end up in caesarean section however it was not statistically significant (p value 0.077) when adjusted with other factors in a regression model. This may be due to the fact that the high risk population either get delivered before 37 weeks or had elective CS at term. Different other studies also proved that medical or obstetrical complications increase the risk of CS.^10^,^11^

The nulliparous mothers with singleton pregnancies, delivering at term with vertex presentation in this study had more chances of abdominal deliveries during night. The reason might be presence of comparatively less experienced on floor junior consultants/residents who were involved in taking the decisions at night, whereas senior Obstetricians were directly involved in the decision making during day.^12^ These findings raised the need of further studies in detail about the influence of competencies of in-house staff on MOD.

In the current study, the possibility of CS increased four times if the women failed to deliver vaginally within ten hours of labour. This finding is consistent with other studies showing labour dystocia as a cause of Caesarean delivery.^13^,^14^ Caughey et al.^14^ studied different factors affecting dystocia in primiparous women including malpresentation, occipitoposterior position, macrosomia etc. which was beyond the scope of this study, however this can be considered for future research.

There would be a better chance of having a vaginal delivery, as the weight of baby increases. It is contrary to what was previously reported and expected. The other available studies proved that the frequency of LSCS increases steadily with increase in birth weight of the baby.^15^,^16^ The birth weights in this study ranged from 2.2-4.8Kg. These finding suggested that more of low birth babies were delivered during this time period, increasing the chances of CS as stated by Sharma et al. (2009) and Patel et al. (2005), that extremes of birth weights are associated with the increased risk of having emergency CS.^1^,^17^ However, further work is required to verify this relationship.

### Limitations of study

This was a retrospective study conducted in one centre only.

## CONCLUSION

The induction of labour, delivering at night time, prolonged labour and birth weight <3kg were found to be associated with the increase CS rate among NTSV deliveries. Therefore further research is mandatory in order to address these factors and to reduce the rate of alarmingly increased abdominal deliveries.
